# Fatigue in fibromyalgia: a conceptual model informed by patient interviews

**DOI:** 10.1186/1471-2474-11-216

**Published:** 2010-09-20

**Authors:** Louise Humphrey, Rob Arbuckle, Philip Mease, David A Williams, Bente Danneskiold Samsoe, Claire Gilbert

**Affiliations:** 1 Mapi Values, Adelphi Mill, Bollington, Macclesfield Cheshire SK10 5JB, UK; 2 Seattle Rheumatology Associates, Seattle, Washington, USA; 3 Chronic Pain and Fatigue Research Center, University of Michigan, 24 Frank Lloyd Wright Drive, Ann Arbor, MI 48106, USA; 4 The Parker Institute, Frederiksberg Hospital, Copenhagen University Hospital, Nordre Fasanvej 57, DK-2000 Frederiksberg, Denmark; 5 Pfizer Ltd, Sandwich, Kent, CT13 9NJ, UK

## Abstract

**Background:**

Fatigue is increasingly recognized as an important symptom in fibromyalgia (FM). Unknown however is how fatigue is experienced by individuals in the context of FM. We conducted qualitative research in order to better understand aspects of fatigue that might be unique to FM as well as the impact it has on patients' lives. The data obtained informed the development of a conceptual model of fatigue in FM.

**Methods:**

Open-ended interviews were conducted with 40 individuals with FM (US [n = 20], Germany [n = 10] and France [n = 10]). Transcripts were analyzed using qualitative methods based upon grounded theory to identify key themes and concepts.

**Results:**

Participants were mostly female (70%) with a mean age of 48.7 years (range: 25-79). Thirty-one individuals (i.e., 77.5%) spontaneously described experiencing tiredness/lack of energy/fatigue due to FM. Participants discussed FM fatigue as being more severe, constant/persistent and unpredictable than normal tiredness. The conceptual model depicts the key elements of fatigue in FM from a patient perspective. This includes: an overwhelming feeling of tiredness (n = 17, 42.5%), not relieved by resting/sleeping (n = 15, 37.5%), not proportional to effort exerted (n = 25, 62.5%), associated with a feeling of weakness/heaviness (n = 20, 50%), interferes with motivation (n = 22, 55%), interferes with desired activities (n = 27, 67.5%), prolongs tasks (n = 15, 37.5%), and makes it difficult to concentrate (n = 21, 52.5%), think clearly (n = 12, 30%) or remember things (n = 9, 22.5%).

**Conclusion:**

The majority of individuals with FM who participated in this study experience fatigue and describe it as more severe than normal tiredness.

## Background

Fibromyalgia (FM) is a chronic disorder characterised by widespread pain and tenderness [1,2], with an estimated prevalence in adults ranging from 0.5-10% worldwide [3-9] with a predominance among females [10]. In addition to chronic widespread musculoskeletal pain, symptoms of fatigue, sleep disorders, headaches, memory or concentration problems, mood disturbances, and stiffness are also commonly associated with FM [1,2,4,11].

Previous focus groups found that, in addition to pain and sleep disturbance, fatigue was perceived by patients to be one of the three most bothersome symptoms of FM [11]. A Delphi study by 23 FM expert clinicians identified fatigue as being the second most important domain to measure (after pain), and it was subsequently rated as third most important (after pain and overall FM) by a Delphi study involving patients [12]. At the Outcome Measures in Rheumatology (OMERACT) 7 workshop composed of clinicians, researchers, regulatory and industry representatives, 94% of participants agreed that fatigue was an essential domain to measure in FM clinical trials [13]. Thus, there is growing consensus among patients, as well as clinical experts, that fatigue is a key symptom to assess in clinical trials of FM. This is further supported by other publications of qualitative FM patient research [14-18].

Like pain, the measurement of fatigue relies on patient report. Recent guidance from regulators highlights that patient input (ideally in the form of qualitative research) is critical when developing or selecting a patient-reported outcome (PRO) measure for a specific condition [19,20]. Existing fatigue measures such as the Multidimensional Fatigue Inventory (MFI) [21] and the Multidimensional Assessment of Fatigue (MAF) have been used extensively in FM clinical trials [22-27]. However, no existing measures were developed with FM specifically in mind and qualitative interviews with FM patients were not included in the development of any existing fatigue measures. Thus, in-depth, qualitative research with FM patients is needed that focuses on exploring the experience of fatigue in FM in order to provide an understanding of how it can be best measured (which may be through using existing instruments or through the development of a new, FM-specific measure of fatigue).

The current project seeks to explore fatigue in detail through in-depth, qualitative interviews with individuals who have FM. The results will inform the development of a conceptual model of fatigue in FM. The conceptual model will define the properties of FM fatigue (and the relationship between each concept and the symptom) and will then be used to support the selection or development of PROs for this symptom.

## Methods

### Sample and study design

Open-ended, qualitative interviews were conducted with 40 individuals with FM in the US (5 male, 15 female), Germany (5 male, 5 female) and France (2 male, 8 female) (Table 1). Participants were recruited through primary care physicians/general practitioners, pain specialists and rheumatology specialists and the sample included individuals from three different countries/cultures/languages to increase the likelihood that findings could be generalized across cultures/languages. Purposive sampling ensured the inclusion of individuals from a range of educational backgrounds and ethnicities [28]. In each country, a minimum of 20% of participants were male, and a minimum of 30% had an education level of high school (or equivalent) or less. In the US, a minimum of 10% were non-Caucasian to ensure ethnic diversity.

**Table 1:** Demographic characteristics of the sample.

Subject Characteristics	US (n = 20)	Germany (n = 10)	France (n = 10)	Total (n = 40)
**Age (years)**				
Mean	49.4	52.4	43.4	48.7
Median	50	51	41	49
Min, Max	25,69	46,58	30,58	25,69
**Gender % (n)**				
Male	25 (5)	50 (5)	20 (2)	30 (12)
Female	75 (15)	50 (5)	80 (8)	70 (28)
**Current Living status % (n)**				
Live alone	15 (3)	80 (8)	50 (5)	40 (16)
Live with husband/wife/partner	30 (6)	20 (2)	50 (5)	32.5 (13)
Live with parents/family or friends	45 (9)	0	0	22.5 (9)
Other	10 (2)	0	0	5 (2)
**Ethnicity % (n)**				
Hispanic	0 (0)	0	N/A	0
Caucasian	80 (16)	90 (9)	N/A	83.3 (25)
African American	10 (2)	0	N/A	6.7 (2)
Asian Oriental or Pacific Islander	0 (0)	0	N/A	0
Other	10 (2)	10 (1)	N/A	6.7 (2)
**Highest Education Level US and Germany % (n)**				
Secondary school education or less	20 (4)	20 (2)	N/A	20 (6)
Vocational school or some college	30 (6)	40 (4)	N/A	33.3 (10)
University/College degree	45 (9)	20 (2)	N/A	36.7 (11)
Post-graduate degree qualification	5 (1)	20 (2)	N/A	10 (3)
				
**Highest Education Level France % (n)**				
Secondary school education or less	N/A	N/A	40 (4)	40 (4)
More than High School (France)	N/A	N/A	60 (6)	60 (6)
**Do you currently work in a paid capacity (full or part-time) % (n)**				
Yes	65 (13)	20 (2)	30 (3)	45 (18)
No	35 (7)	80 (8)	70 (7)	55 (22)

### Inclusion/exclusion criteria

Participants were at least 18 years of age, met the American College of Rheumatology (ACR) criteria for FM (1) and were willing and able to participate in a 90-minute interview. Participants were excluded if they had significant physical or psychiatric co-morbidities (including severe pain not related to FM) that might have interfered with their experience of, or ability to talk about, FM. Of note, participants were not required to have fatigue - they were only required to have a diagnosis of FM because this study was part of a larger project looking at other FM domains in addition to fatigue.

### Ethics

Ethics approval of the study protocol, documents and procedures was granted by Copernicus, a US centralized Independent Review Board, and written informed consent was obtained from all participants prior to entry into the study.

### Interview methods

All interviews were conducted by an experienced qualitative interviewer, who was a native speaker of the language in which the interview was performed. Using a semi-structured interview guide, questions were initially open-ended to ensure participants were not biased by the topics of interest (e.g., "tell me about your experience of having fibromyalgia?"). Direct questioning was only used when topics of importance did not arise in response to the open-ended questions.

Participants were first asked to talk about their general experience of FM and then asked to describe good and bad days with FM. Participants were also asked what three things bothered them most about having FM. These exploratory questions were followed by two creative tasks that encouraged participants to talk in a spontaneous, more creative manner about their FM. First, prior to interview, participants were asked to create a drawing or collage representing their FM which was discussed during the interview. In the second task, participants were asked: "if your FM was an animal, what animal would it be?" Participants were asked open questions about "FM" generally rather than fatigue specifically so that if fatigue was discussed, it was identified spontaneously by the participant indicating the importance of the symptom to FM patients.

Only after these open-ended questions and creative tasks did the interview progress to more focused questions relating to FM fatigue (if not already discussed). This methodology gives participants the opportunity to mention FM fatigue spontaneously and also to mention concepts/symptoms/domains of importance which may not have been in the discussion guide [29].

At the end of each interview, participants were asked to list the symptoms of FM they experience and to rate their pain, fatigue, cognition and functioning using Visual Analogue Scales (VAS) and Numerical Rating Scales (NRS). Each interview lasted approximately 90 minutes. Revising the guide was considered after the first five interviews; no revisions were required.

### Patient-reported questions and demographics

As well as completing the VAS and NRS symptom questions, participants also provided information regarding their age, gender, number and age of children, living situation (e.g. living alone, with a partner, etc), highest level of education, work status and ethnicity. The French participants were not asked for ethnicity as it is considered culturally inappropriate.

### Collection of clinician-reported information

To verify the patient-reported data and to provide additional clinical information, the recruiting physicians were asked for background clinical information including: year of diagnosis, current FM treatments and details of co-morbidities. The clinicians chose which of a list of 20 'symptoms and syndromes' commonly associated with FM (e.g. fatigue, stiffness, depression) were present in their patient. There was also space for the clinician to add any other symptoms/syndromes not listed.

### Qualitative analysis

All interviews were audio-taped and transcribed verbatim with any identifiable information removed during transcription. Qualitative analysis of the verbatim transcripts was conducted in the original language version of the transcript by an analyst who was also a native speaker. Coding of quotes by concept/domain involved assigning appropriate codes to patient statements determined by the underlying concept such as 'difficulty remembering' and then grouping concepts into domains, for example, 'cognitive limitations'. This analysis approach is based on a Grounded Theory approach [30-33] and Atlas Ti software (Atlas.ti GmbH Berlin, Germany, version 5.2) was used. The French and German transcripts were coded in both their original language and their English translation (to retain cultural meaning and identify any language subtleties), and the developed code list compared with codes from the US interviews to identify consistency in the concepts emerging from the analysis. The coding scheme, driven by the participants' experience was developed iteratively, changed dynamically as more data was analysed. The reliability of codes and definitions were confirmed through extensive discussion and consensus among analysis team members. Codes were then organised into higher order domains/concepts.

### Saturation

Analysis was conducted to determine whether conceptual saturation was achieved. Saturation has been defined as the point at which no new concepts or sub-concepts emerge with the addition of more interviews [31,34]. In the current research, saturation in the US sample was examined by comparing the first 10 transcripts with the next 10 to identify if any concepts arising in the second set of 10 interviews had not arisen in the first 10 interviews. If new concepts had emerged, additional interviews would have been conducted and the saturation checking process repeated. For country-level saturation, the concepts arising from the French and German transcripts were compared to those arising from the US interviews.

## Results and Discussion

### Background characteristics of the sample

Consistent with the sampling targets and prevalence of FM [10], 28/40 participants (70%) were female, mean age was 48.7 years (range: 25-69 years old) and patients had a range of education levels. Of the 30 participants who provided ethnicity information, the majority were Caucasian (n = 25, 83%).

### Clinician-reported background clinical data

Participants had been diagnosed with FM for a mean of 6.6 years (SD = 5.20, range = 1-18 years). The most common clinician-reported FM-related "symptoms and syndromes" were fatigue (n = 40, 100%), stiffness (n = 39, 98%), sleep disorders (n = 37, 93%), joint aches (n = 36, 90%) and tenderness to touch (n = 34, 85%). Depression was identified in 37.5% (n = 15). Moreover, depression was present among French (n = 7, 70%) and US (n = 8, 40%) participants but was not reported for any German participants. With the exception of depression, there were no patterns of particular symptoms/syndromes being more frequently reported for any one country sample. The majority of participants (n = 30, 75%) had no co-morbidities unrelated to FM.

### Patient-reported questions

**Figure 1:**
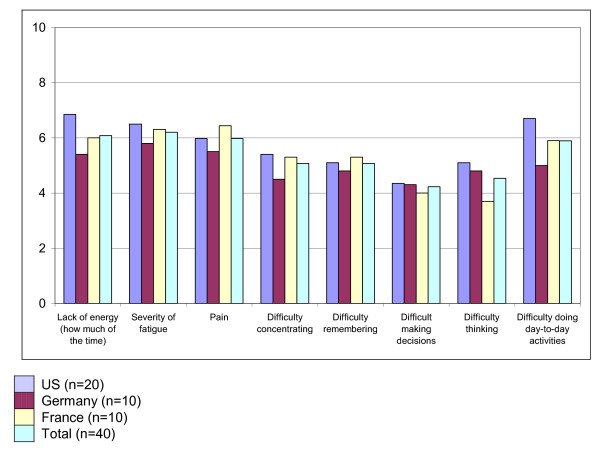
**Patient-reported VAS and NRS items asking about pain, energy fatigue and impact of FM on cognitive functioning and ability to do day-to-day activities (n = 40)**.

Mean scores for the quantitative patient-reported questions are presented in Figure 1. The total mean pain severity score (rated on a VAS) was 5.97, with no major differences among countries. The remaining PRO items used a 0-10 NRS scale with 10 indicating greater difficulty/severity. For all of the NRS items, the mean scores were between 4 and 6 and there were no notable differences among the country samples for most items.

### Qualitative interview findings: experience of fibromyalgia

When asked, "tell me about your experience of FM", pain was the most common spontaneously reported concept (n = 31, 78%), followed by fatigue (n = 17, 43%), sleeping problems (n = 7, 18%) and mobility problems (n = 4, 10%). Participants were asked to state the three things that bothered them the most about having FM. Of the 35 who answered the question, 31 (89%) mentioned pain. The second most commonly mentioned concept was fatigue/tiredness/lack of energy mentioned by 18/35 (51%) participants (11/17 in the US; 2/10 in Germany; and 5/8 in France). The next most commonly mentioned concept was functional limitations arising from FM, mentioned by 9/35 (26%) participants.

Pain and tiredness/fatigue were the most common concepts/themes described when participants discussed their homework collages/drawings. Pain-related concepts were mentioned by 18/40 (45%) participants who chose/drew pictures of people in pain or indicated on their collage an image that represented pain. Fatigue/tiredness was mentioned by 10/40 (25%) - nine of whom were from the US (and one from France). These participants chose/drew pictures that depicted for example, people sleeping, animals carrying heavy loads and characters walking up steep hills. No German participants mentioned fatigue or tiredness in the spontaneous discussion of their homework as they focused primarily on pain. In the second creative task, participants had to think of an animal that represented their FM and what their reasons were behind their chosen animal. Of the 38 participants who completed the task, 20 (53%) chose an animal that related to fatigue/tiredness e.g. sloth or tortoise because these animals were considered 'slow' or 'lazy'. Fourteen (37%) chose an animal that related to pain (e.g. porcupine because of their spines, a lion because a roar symbolized the pain they were in), and six (16%) chose an animal related to the unpredictable and unpleasant nature of FM (e.g. snake 'because you never know when it is going to strike'). Of note, some participants chose more than one animal (e.g. one to represent pain and one to represent fatigue/tiredness) or also referenced more than one concept when explaining their choice of animal.

### Qualitative interview findings: patient experience of fibromyalgia fatigue

**Figure 2:**
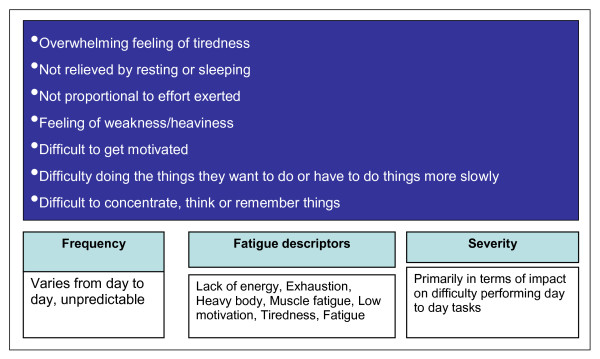
**Conceptual model of fibromyalgia fatigue**.

A conceptual model of FM fatigue was developed based on the qualitative findings and on the previous qualitative research that was reviewed [11,12,14,15] (Figure 2). FM fatigue was described as an overwhelming feeling of tiredness that was not relieved by sleep or rest and is often not in proportion to the effort exerted (i.e. participants described becoming tired after doing very little). Many described their fatigue as 'feeling weak' or their body feeling heavy and almost all participants talked about having to force themselves to do things or described having difficulty getting motivated to do things. Participants differentiated between FM fatigue and normal tiredness by referring to the fact that FM fatigue limited them in doing daily activities or caused difficulty concentrating, thinking clearly and/or remembering things. Detailed findings to support each of these FM fatigue characteristics are provided in Table 2, each with sample participant quotes.

**Table 2:** Summary of the characteristics that define FM fatigue

FM-fatigue characteristics	US (n = 20)	France (n = 10)	Germany (n = 10)	Total (n = 40)	Example Quote
Overwhelming feeling of tiredness	10	4	3	17	*"Feeling of just being, just overwhelmed by tired - just being tired and sleepy and fatigued" *- Female, aged 61, US *"you're that tired, it's this big oppressive - it's like a blanket and it wraps you up...it's overwhelming" *- Female, aged 33, US
Not relieved by resting or sleeping	10	1	4	15	*"I could sleep 20 hours and still be tired. That is terrible." *- Female, aged 58, Germany *"But when you sleep, it doesn't resolve it. You still wake up tired. " - *Female, aged 50, US
Not proportional to effort exerted	17	4	4	25	*"...whereas in this case you've done nothing, you shouldn't feel tired, but you do, you're weary, completely lethargic." *- Female, aged 39, France *"In general, the fatigue, for me, it strikes quite bad, because I normally like an active person. And now to do the simplest little thing, I can be where I'm just totally worn out. Like I've been doing something all day long very strenuous." *- Female, aged 49, US
Feeling of weakness or heaviness	10	4	6	20	*"your whole body feels really heavy, like I was saying about the cement suit thing. I just feel like there's a real heaviness to it." *-Female, aged 41, US *"I don't shop, my friends do it for me, it is enough to go with them and say what I want; that is exhausting enough. Exhaustion means that my body feels lifeless and weak." *- Female, aged 50, Germany
Difficult to get motivated	12	5	5	20	*"...you just don't have the energy and the motivation to get out of the bed and do anything" *- Male, aged 50, US *"Yes, well, the lack of enthusiasm which I have, that I have to overcome to motivate myself into doing something." - Female, aged 58, Germany*
Difficulty doing the things they want to do	12	3	9	24	*"...so you are too tired to go shopping, for example" - Male, aged 49, Germany*
Having to do things more slowly	8	4	3	15	*"Because I'm sluggish no matter what I'm doing, that's it! I need more time doing things now that took me 5 minutes before!" - Female patient, aged 30, France*
Difficult to concentrate, think or remember things	12	7	8	27	*"Well I think that the fatigue causes - I think it causes a lot of things to be missing in a person's concentration." *- Female, aged 56, US *"...you can't think and you can't hear, and everything's grey." *- Female, aged 33, US

#### Overwhelming feeling of tiredness

Seventeen participants (43%) described how at times they would become overwhelmed by their fatigue (15 spontaneously and two when probed). Eight of these participants (20% of the total sample) were, at times, overwhelmed to the point that they were unable to do anything.

#### Not relieved by resting or sleeping

Fifteen participants (38%) described still experiencing tiredness or fatigue, even after a good night's sleep.

#### Not proportional to effort exerted

Twenty-five participants (63%) talked about becoming tired very easily. For example, some individuals talked about being exhausted after doing hardly anything.

#### Feeling of weakness or heaviness

Eleven participants (28%) talked about their body feeling heavy, weak or not having any strength. Many of these individuals felt so weak that it was difficult to do daily activities.

#### Difficult to get motivated

Thirty-three participants (82.5%) talked about fatigue as not having the motivation to do things or having to force themselves to do things. Sixteen (40%) also discussed the concept of motivation in relation to having difficulty getting out of bed in the morning or 'getting going' in the morning. Others discussed the amount of effort required to do things or of needing to force themselves to do things.

#### Difficulty doing the things they want to do

The majority of participants (24/40, 60%) across all three countries spontaneously talked about fatigue/tiredness making it difficult to do the things that they want or need to do; one further individual talked about this when probed. Activities affected by fatigue included strenuous physical activities (e.g. playing sports, yard-work), cognitive tasks (e.g. paying bills) and simple self-care activities (e.g. pouring a cup of coffee, getting dressed).

#### Having to do things more slowly

Fifteen participants (38%) talked specifically about having to do things slowly, or taking longer to do things due to tiredness or fatigue. In some cases this seemed to be related to the feeling of heaviness or weakness.

#### Difficult to concentrate, think or remember things

Many participants (n = 27, 68%) talked about a mental or cognitive component to their fatigue. Twenty-one participants (53%) talked about fatigue/tiredness affecting their ability to concentrate, nine (23%) talked about difficulty remembering things, 12 (30%) talked about having difficulties thinking clearly and eight (20%) mentioned having trouble staying focused. As examples of activities affected by cognition problems some referred to paperwork, others described having difficulty holding simple conversations.

#### Terms used to refer to fatigue/tiredness/exhaustion

We were interested to know if participants distinguish between "fatigue" that might result from FM and normal tiredness. We were also interested to know if participants use the term "fatigue" to refer to something more severe than normal tiredness. Throughout the interviews, participants spontaneously used many different terms to describe their FM-related fatigue/tiredness. Table 3 summarizes the terms participants used most commonly to describe fatigue. At some point in the interview almost all participants talked either of 'feeling tired' (n = 27, 68%), being 'fatigued' (n = 14, 35%), having 'a lack of energy'/'no energy'/'little energy' (n = 20, 50%) or feeling 'exhausted' (n = 13, 33%). Some talked about feeling 'worn out' (n = 12, 30%) or 'feeling weak', (n = 11, 28%); others talked about feeling 'overwhelmed' (n = 15, 38%) by their fatigue. In the French sample, 'fatigué' was used by all participants to refer to their tiredness/fatigue. This term is usually translated as meaning 'tired' or 'tiredness', but in some contexts could also be translated as meaning 'fatigue' - we have only translated it as meaning tired. Also of note, in German there is a word that is typically translated as meaning 'tired/tiredness' and another word translated as 'exhausted'. However, no word that could be directly translated as meaning 'fatigue', distinct from these two terms was referred to. In the German sample 'tired' was by far the most common term.

**Table 3:** Summary of terminology used spontaneously to describe FM fatigue

Term	US (n = 20)	France (n = 10)	Germany (n = 10)	Total (n = 40)	Example Quote
Tired/Tiredness	19	3	5	27	"Tired - tiredness is, I'm tired but I'm still going about my business, and when I get extremely tired, I just know you have to stop and you need to rest, lay down." Female, aged 69, US
Tire easily	17	4	4	25	"This morning I got up and I was a little slow getting up and getting moving. So I thought, OK, it's going to be one of those days. But then I'm sitting there, after my husband goes, OK, feeling better. And then the tiredness came in. It's like, no, you're going to have to crash, that's it." Female, aged 49, US
Tired upon waking	5	2	2	9	"And then okay... every day, every day... being tired when you wake up... I say, "After all, you didn't do anything excessive, nothing excessive, you..." ...I have a relatively healthy lifestyle... so.. I don't see the why and how of it...!" Male, aged 47, France
Sleepiness	5	0	1	6	Interviewer: "So, can you just describe that feeling, the tiredness feeling." Patient: "Sometimes it's a sleepiness, as in sitting in my recliner on a bad day, I'll fall asleep." Female, aged 49, US
No energy/Lack of energy	16	0	4	20	"Just very tired, and just - really no energy, energy level very low." Female, aged 50, US
Fatigue^a^	14	N/A	N/A	14	"The fatigue, it's number one, because I can deal with the pain, at least up to a certain point, but the fatigue there's nothing you can do besides sleep. There is no way to help that. There's no pill you can take, there's no medicine." Female, aged 33, US
Feeling drained	6	4	1	11	"Fatigue is just always just drained. Like feeling drained." Female, aged 34, US
Feeling weak	5	2	4	11	"I feel like my body doesn't have energy. Like I'm sitting here and the effort that it would take for me to get up and walk, or run, I would think about it before a normal person would, I think. I would think about the energy it takes, and I would feel - I just feel like weighted down. That's what the fatigue feels like. It's almost a weak feeling, or a heavy feeling." Female, aged 25, US
Exhausted/ exhaustion	8	2	3	13	"I would feel tired, or exhausted, or just run down, those kind of terms, which is all connected." Female, aged 50, US
No get up and go	5	0	0	5	"It feels like it's like - like a plug has been pulled. The fatigue does, feels like a plug has been pulled. And your get up and go, your energy has just drained away. That's the best I can describe it." Male, aged 43, US
Shattered	0	0	5	5	"How can I describe it? I don't have any energy, I don't feel so tired that I have to lie down but I am shattered. I have no desire to get up, I stay sitting down and wait for the next burst of pain." Male, aged 52, Germany
Worn out	1	6	5	12	"And now to do the simplest little thing, I can be where I'm just totally worn out. Like I've been doing something all day long very strenuous." Female, aged 49, US
Overwhelmed	8	4	3	15	"When tiredness happens, there's no relief. It's like an overwhelming, overarching, penetrating, consuming tiredness." Female, aged 61, US
Tired even after resting/good night's sleep	10	1	4	15	"But when you sleep, it doesn't resolve it. You still wake up tired. So in turn, it affects your motivation, because you have things that you want to get done." Female, aged 50, US
Emotional tiredness	1	0	0	1	"So there's an emotional tiredness that comes with it. And I guess that would be the difference." Male, aged 43, US

^a ^In French and German there is no term for fatigue that is distinct from the terms that are used for tiredness and exhaustion

Different participants often used the same term to refer to different things; for example some participants considered 'energy' to refer to the mental component of fatigue, while others related 'energy' to the physical component of fatigue. Furthermore, many participants seemed to use the different terms interchangeably and understood them all to be related to the same concept - for example, when describing their tiredness participants would often describe it as "a lack of energy'. Nevertheless, whichever words participants used to talk about their fatigue, all agreed it was worse than normal tiredness and referred to the characteristics in the model presented in this study.

### Qualitative interview findings: relationship between fatigue and sleep problems

Almost all participants (n = 38, 95%) reported sleep problems due to their FM, most commonly because of pain. Participants were asked whether or not the fatigue/tiredness they experienced was related to sleep problems. Four concepts emerged: 1) the quality of sleep was perceived by participants as impacting the severity of fatigue the following day; 2) fatigue however, often occurred irrespective of sleep quality; 3) difficulty sleeping occurred irrespective of how tired a person was; and 4) some individuals experienced daytime sleepiness; however most distinguished daytime sleepiness from FM-fatigue/tiredness. These findings suggest that while sleep problems and fatigue are related, the experience of FM-fatigue was not perceived as being entirely explained by the quantity or quality of sleep.

### Qualitative interview findings: saturation analysis

In the US sample, saturation of all concepts was achieved in the first 10 US interviews - no new concepts or terms for talking about fatigue arose from the second 10 US interviews that had not arisen in the first 10 interviews. Country-level saturation was also attained across the three samples - no new fatigue sub-concepts emerged from the analysis of the French and German transcripts that had not been mentioned in the US interviews.

### Qualitative interview findings: gender and country sub-group analysis

Analysis of gender differences was conducted to examine the similarities/differences in the way men and women talked about FM and their fatigue. Men and women described the fatigue experience and its impact in much the same way. There was some evidence that men focused more on pain associated with their FM, whereas women talked more about fatigue. Analysis of any differences by country/language was also conducted; although fatigue was frequently and spontaneously reported in all three countries, US participants talked the most about their fatigue, followed by the German sample. French participants talked about fatigue the least. However, all concepts included in the model were mentioned by participants in all three countries, and were clearly pertinent across all country samples. Participants from all countries consistently talked about the impact on daily activities or difficulties with concentration, thinking clearly and/or remembering things as being delineating factors between FM fatigue and "normal" tiredness.

In addition, the clinician-reported data showed that more French and US patients experienced depression than German patients. Although depression and fatigue is strongly linked [35], our analysis revealed that there were no sub-concepts of fatigue or FM impact that were mentioned by only depressed patients. This suggests that the concepts identified by participants in this study were relevant for all and not just those who had depression.

The primary objective of this research was to better understand the patient experience of FM fatigue through open-ended, qualitative research, as advocated by regulators and experts in PRO development [19,36,37]. Findings suggest that, while widespread pain is the primary symptom of FM, fatigue appears to be the second most important symptom and one that has a considerable impact on patients' lives. Although fatigue was not an inclusion criterion in the present study, all participants experienced fatigue. Fatigue was often one of the first problems that participants mentioned when asked open-ended questions. In some cases participants described their fatigue as being worse than their pain because of associated daily activity limitations. These findings are consistent with, and build upon, previous exploratory focus groups, which highlighted the importance of fatigue in FM [11]. In the focus groups, fatigue was described as one of the worst symptoms of FM and was seen as a constant presence that necessitated pacing the activities of their lives. This theme is consistent with the findings in the present research where participants reported having to do things more slowly due to their fatigue. In an earlier focus-group, participants also complained about a lack of energy that accompanied an overwhelming feeling of constant fatigue, consistent with this research. Thus, the current research is consistent with the previous research into FM fatigue [5,11,14-18] and provides strong evidence that the majority of individuals with FM experience fatigue which has a substantial impact on daily activities and wellbeing.

The finding that men seemed to focus more on pain, whereas women talked more about fatigue, may indicate that it is more socially acceptable for women to report fatigue. Further investigation of this gender difference is warranted. The reasons for the differences between the three countries in the frequency of fatigue being reported are unclear, and caution must also be applied in drawing conclusions from a small qualitative sample. The differences could be due to: (1) country differences in fatigue being a recognized symptom of FM; (2) language differences (in German and French there is only one word for both fatigue and tiredness - the absence of a 'clinical' sounding word such as fatigue might make patients more reluctant to report tiredness/fatigue); or (3) cultural difference in willingness to report fatigue. However, a significant proportion of the French and German samples experienced fatigue, and the impact on their lives was similar to that of the US patients. Quantitative studies looking at these differences in reporting fatigue are required to provide an understanding of whether the gender and country differences reported here are replicated in larger samples, or if they are an artefact of this small, qualitative sample [5,38,39].

To build on this current work, it would be interesting to compare the way FM patients talk about fatigue compared to patients with other severe articular diseases (e.g. rheumatoid arthritis), given that both groups of patients report this symptom [40]. A comparative qualitative study with patients who have similar conditions would be warranted to understand these differences and generate a real conceptual view of the specificity of fatigue for these comparable conditions.

The qualitative findings reported here highlight the challenges of developing a measure of fatigue that has strong content validity. Thus, a possible solution to the challenge of measuring FM fatigue might be to ask individuals about all of the different characteristics of fatigue outlined in the model, and to phrase the questions using several of the different descriptors commonly used by patients.

## Conclusions

In conclusion, this study demonstrated that in addition to pain, fatigue was an important symptom for individuals with FM. Individuals with FM used many terms to talk about the concept of fatigue including 'tired', 'fatigue', 'no energy', and feeling 'worn out', or 'exhausted'. There was consensus among participants that FM-related tiredness or lack of energy is worse than normal tiredness. The conceptual model of FM fatigue captures elements that distinguish FM fatigue from normal tiredness from a patient perspective. This conceptual model is currently being used to support the development of a new PRO for fibromyalgia fatigue.

## Competing interests

Ms Humphrey and Mr Arbuckle received research/grant support from Pfizer and were contracted by Pfizer to assist in the present study.

## Authors' contributions

All authors contributed to the study design, provided input into the writing of the protocol, interview guide and study documents, were involved in the interpretation of the results, reviewed the conceptual model and were involved in the drafting of the manuscript. All authors read and approved the final draft.

## Pre-publication history

The pre-publication history for this paper can be accessed here:

http://www.biomedcentral.com/1471-2474/11/216/prepub
